# Duodenal tuberculosis with gastric outlet obstruction: a case report of successful diagnosis and treatment, with review of literature

**DOI:** 10.1186/s40792-024-01840-x

**Published:** 2024-02-15

**Authors:** Nami Sato, Masayuki Shiobara, Kazuo Wakatsuki, Kosuke Suda, Kotaro Miyazawa, Toshiaki Aida, Yoshihiro Watanabe, Katsunobu Tawada, Yoshiki Matsubara, Yohei Hosokawa, Shigeru Yoshioka

**Affiliations:** 1 Department of Surgery, Chiba Kaihin Municipal Hospital, 3-31-1 Isobe, Mihama-Ku, Chiba, 261-0012 Japan; 2 Department of Gastroenterology, Chiba Kaihin Municipal Hospital, 3-31-1 Isobe, Mihama-Ku, Chiba, 261-0012 Japan; 3 Department of Pathology, Chiba Kaihin Municipal Hospital, 3-31-1 Isobe, Mihama-Ku, Chiba, 261-0012 Japan

**Keywords:** Duodenal tuberculosis, Endoscopic ultrasound-guided fine-needle aspiration, Gastric outlet obstruction, Laparoscopic surgery, Mycobacterium tuberculosis

## Abstract

**Background:**

Duodenal tuberculosis (TB) is extremely rare, and its diagnosis is challenging owing to the lack of specific symptoms and radiological or endoscopic findings. When it leads to gastric outlet obstruction (GOO), diagnosing it accurately and providing appropriate treatment is crucial. However, this is often overlooked.

**Case presentation:**

A 35-year-old man presented with abdominal pain, fullness, vomiting, and weight loss. Upper gastrointestinal endoscopy and radiography revealed nearly pinpoint stenosis with edematous and reddish mucosa in the D1/D2 portion of the duodenum. Computed tomography (CT) showed the duodenal wall thickening, luminal narrowing, multiple enlarged abdominal lymph nodes, and portal vein stenosis. Conventional mucosal biopsy during endoscopy revealed ulcer scars. We initially suspected stenosis due to peptic ulcers; however, chest CT revealed cavitary lesions in both lung apices, suggesting tuberculosis. Due to the suspicion of duodenal TB and the need to obtain deeper tissue samples, endoscopic ultrasound-guided fine-needle aspiration (EUS-FNA) was performed. The tissue sample showed caseating granulomas with multinucleated giant cells, and acid-fast bacilli were positive by Ziehl–Neelsen staining. The patient was diagnosed with duodenal TB and subsequent GOO. Because the patient had difficulty eating, surgical intervention was prioritized over antitubercular drugs, and laparoscopic gastrojejunostomy was performed. The patient started an oral diet on the 3rd postoperative day and began antitubercular treatment immediately after discharge on the 11th day. During the 6th month of treatment, endoscopic examination revealed residual duodenal stenosis; however, the bypass route functioned well, and the patient remained asymptomatic.

**Conclusions:**

An aggressive biopsy should be performed to diagnose duodenal TB. EUS-FNA has proven to be a useful tool in this regard. Both nutritional improvement and antitubercular treatment were achieved early and reliably by performing laparoscopic gastrojejunostomy for duodenal TB with GOO.

## Background

According to a World Health Organization report [1], the estimated number of patients with tuberculosis (TB) worldwide in 2021 was 10.6 million, indicating that TB remains a significant infectious disease. Duodenal TB is an extremely rare form of this disease [2]; however, it is crucial to make an early and accurate diagnosis, leading to appropriate treatment. This is because it tends to cause obstructive symptoms as the disease progresses. Improvement in duodenal TB with obstruction can be expected with a combination of antitubercular therapy (ATT) and invasive interventions. However, owing to its rarity, nonspecific symptoms, uncharacteristic endoscopic findings, and low diagnostic rates in pathology and bacteriology, duodenal TB is often overlooked. Consequently, patients may experience periods without proper treatment or undergo unnecessary treatment.

We report a case of gastric outlet obstruction (GOO) caused by duodenal TB. In this case, a successful preoperative diagnosis was made using endoscopic ultrasound-guided fine-needle aspiration (EUS-FNA). Laparoscopic bypass surgery was performed as a minimally invasive and effective treatment for duodenal TB with GOO. This procedure allowed the patient to resume eating and receive appropriate ATT as quickly as possible.

## Case presentation

**Fig. 1:**
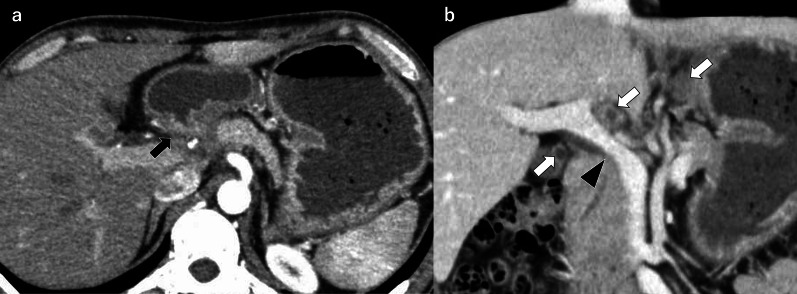
Abdominal computed tomography (CT) images on admission. **a** Duodenal stricture and wall thickening (black arrow). **b** Numerous enlarged abdominal lymph nodes (white arrows). Some of them have low CT values, suggesting necrosis. The portal vein is constricted by a low-density area along the hepatoduodenal ligament (head arrow)

A 35-year-old man with a history of epigastric pain, fullness after eating, and vomiting during the previous month was admitted to our hospital. He had experienced weight loss of 12 (kg) in the past year. He came to Japan from Myanmar four years ago. He had no history of chronic cough, fever, or diseases requiring immunosuppressants, and his family had no history of TB. Blood test results were normal, and anti-human immunodeficiency virus antibodies were negative. Abdominal computed tomography (CT) revealed gastric dilatation, thickening of the duodenal wall with luminal narrowing, and multiple enlarged low-density abdominal lymph nodes. Furthermore, portal vein stenosis was observed due to a low-density area along the hepatoduodenal ligament (Fig. 1a, b).

**Fig. 2:**
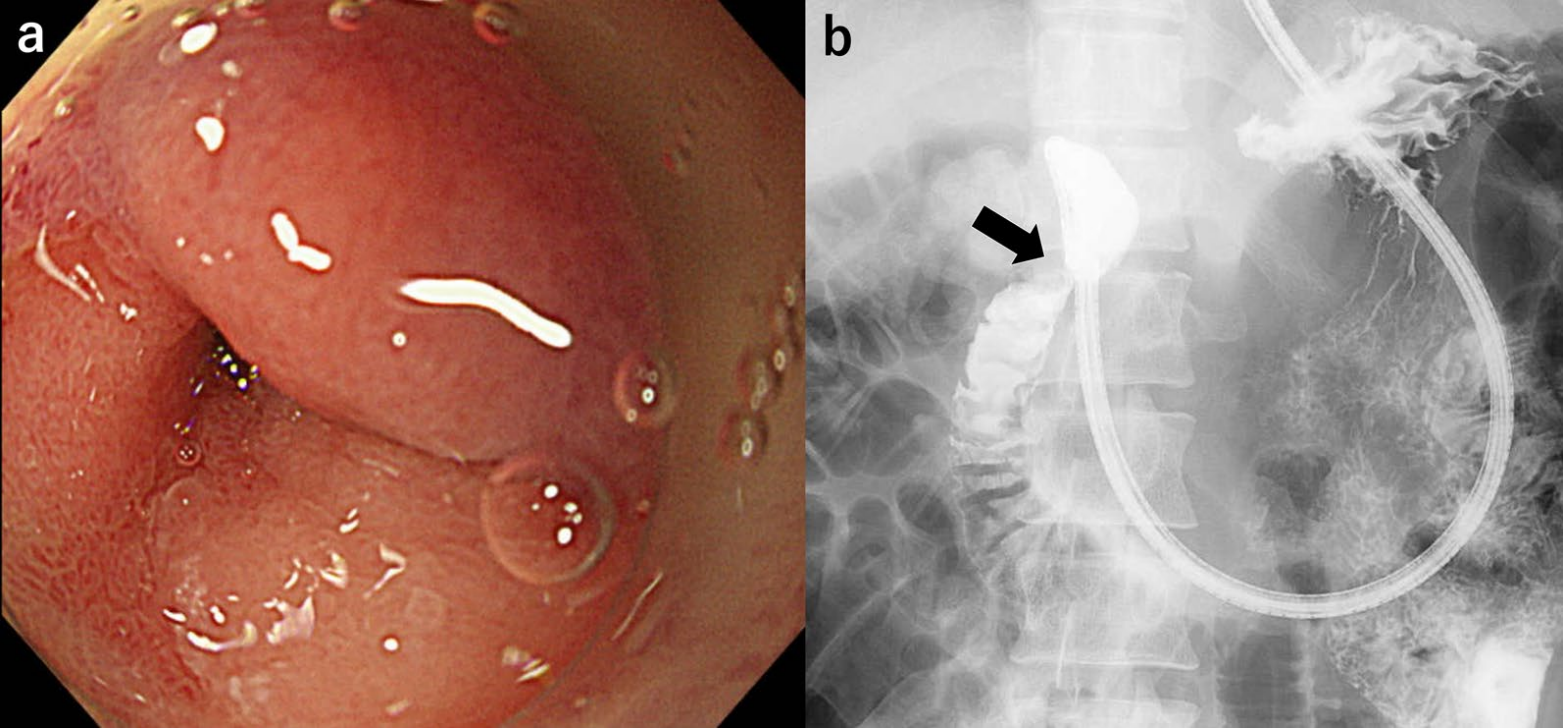
Images of Gastrointestinal (GI) Endoscopy and Upper GI Radiography. **a** Endoscopy showing a duodenal pinhole stricture. The mucosa exhibited edema and redness; however, no clear ulcerations were observed. **b** Upper GI contrast showing a short stricture at D1/D2 of the duodenum (arrow)

The patient underwent an upper gastrointestinal (GI) endoscopy several days after the nasogastric tube placement. Endoscopic examination revealed a near-pinhole stricture in the D1/D2 portions of the duodenum (Fig. 2a). The narrowed area showed an edematous and reddish mucosa and a 5.8-mm-diameter scope could pass through, but a 9.7-mm-diameter scope could not. No distinct ulcers are observed. A biopsy of the mucosa revealed histopathological features consistent with ulcer scars. Upper GI radiography revealed a short stenotic segment in D1/D2 (Fig. 2b).

**Fig. 3:**
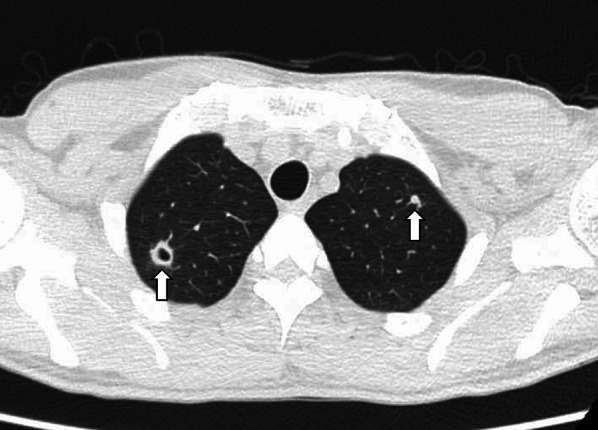
Image of lung computed tomography (CT). Cavitary lesions noted in the apices of both lungs (arrows)

**Fig. 4:**
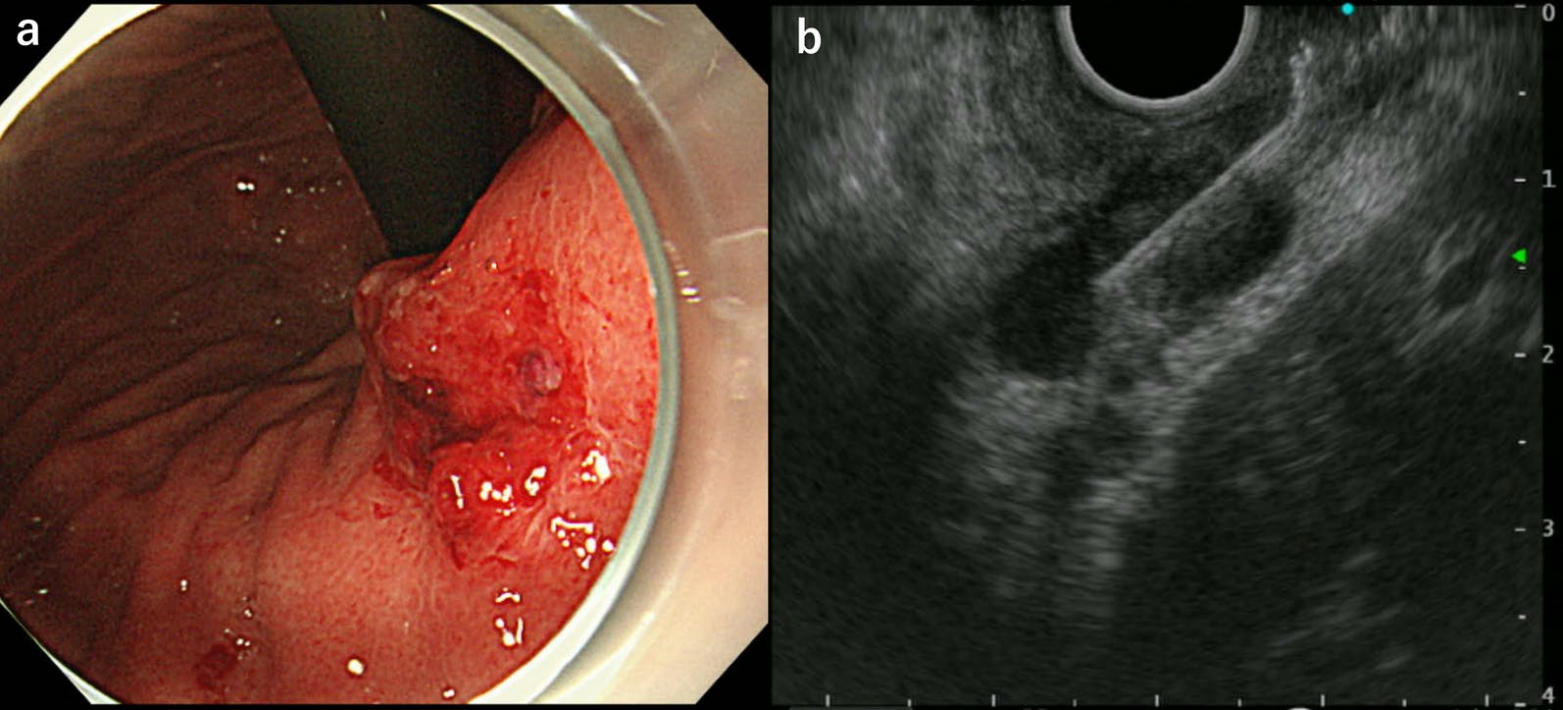
Images of the second gastrointestinal endoscopy and ultrasound endoscopy. **a** An ulcerative lesion is found on the lesser curvature of the upper body of the stomach. **b** Endoscopic ultrasound-guided fine-needle aspiration was performed on lymph nodes contiguous with a gastric ulcer

**Fig. 5:**
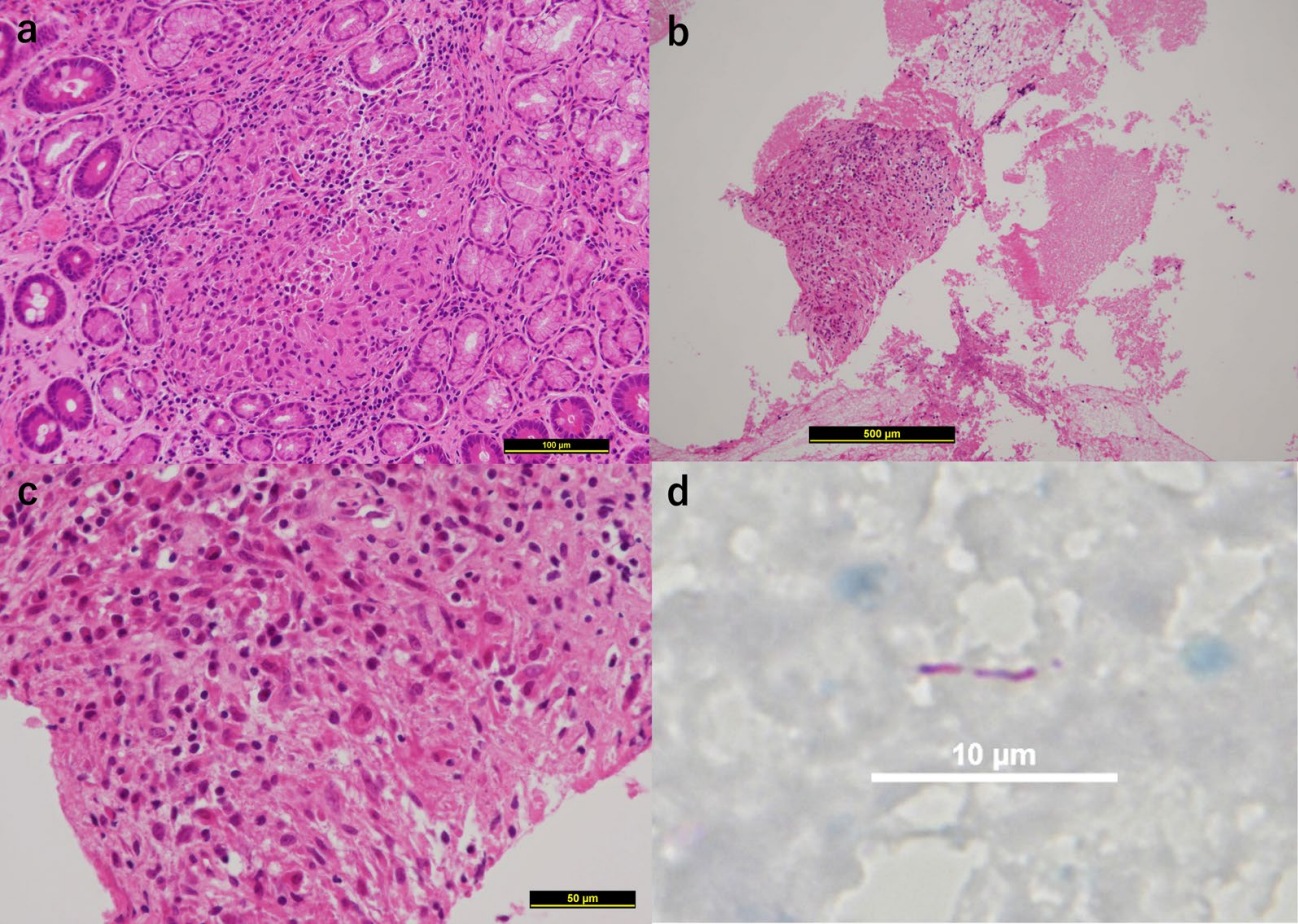
Pathological findings in biopsied tissue. **a** Epithelioid granuloma in duodenal mucosal tissue. **b** Granuloma with caseous necrosis in the lymph node obtained by EUS-FNA. **c** Enlarged image of **b**. Epithelioid granuloma with multinucleated giant cells. **d** Ziehl–Neelsen staining of lymphoid tissue was positive for mycobacteria

Our initial impression was duodenal stenosis caused by peptic ulcers. Therefore, intravenous omeprazole therapy was initiated immediately after admission. However, chest CT revealed cavitary lesions in both the lung apices (Fig. 3). We suspected tuberculosis and believe that there is a need for further detailed investigation of the abdominal lesions. A QuantiFERON-TB Gold (QFT) blood test revealed a positive result. However, sputum and gastric juice samples were negative on smear microscopy, acid-fast bacilli cultures, and *Mycobacterium tuberculosis* polymerase chain reaction (PCR) tests. A second-look endoscopy showed no improvement in duodenal narrowing despite the continuous administration of omeprazole. We identified a new ulcerative lesion in the lesser curvature of the upper gastric body (Fig. 4a) and performed multiple mucosal biopsies. Concurrently, we planned EUS-FNA to obtain biopsies from deeper layers. EUS revealed that the lymph nodes adjacent to the gastric wall had penetrated and formed a gastric ulcer. Therefore, EUS-FNA was performed (Fig. 4b). Unfortunately, the duodenum could not be observed using EUS because it was too narrow to allow scope insertion. Histopathological examination revealed granulomatous inflammation in the biopsied tissues of the duodenal mucosa and gastric ulcers (Fig. 5a). Furthermore, granulomatous inflammation with caseating necrosis and multinucleated giant cells were identified in the lymph node tissue obtained from EUS-FNA (Fig. 5b, c). Ziehl–Neelsen staining confirmed the presence of acid-fast bacilli (Fig. 5d); however, the bacterial culture did not confirm the presence of tuberculous bacilli.

**Fig. 6:**
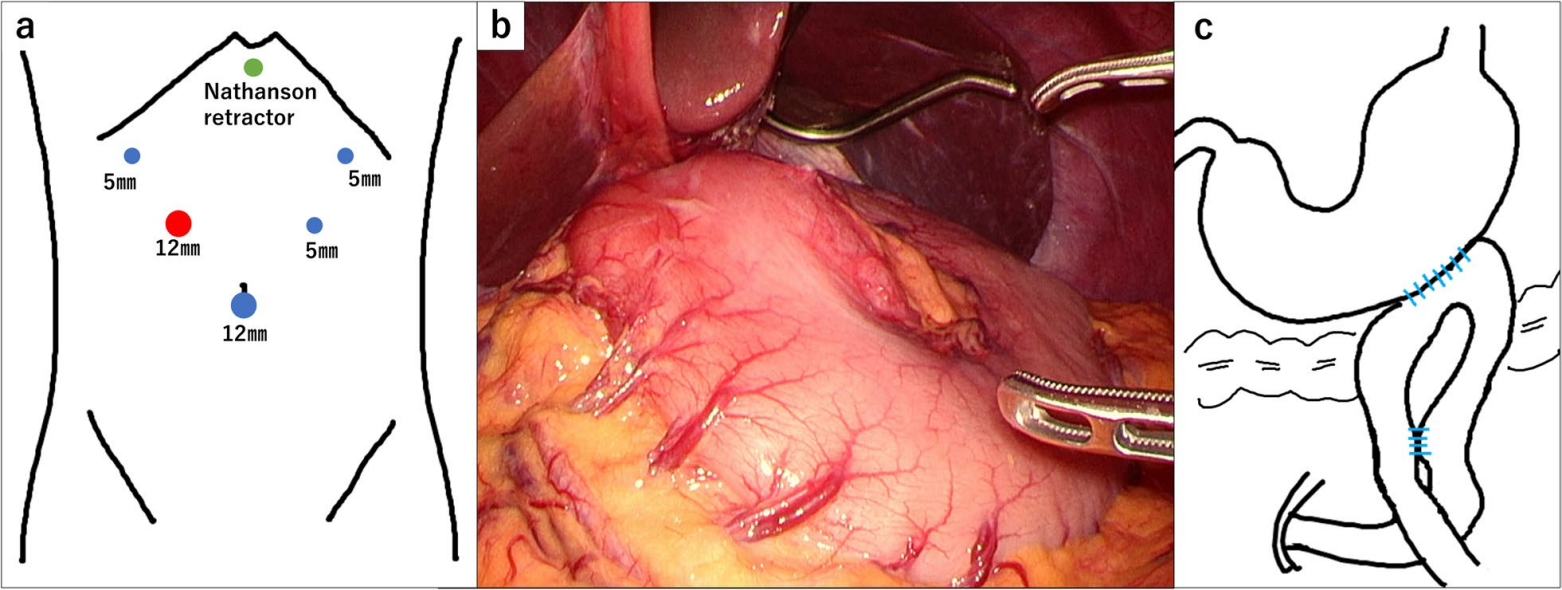
Images of surgical procedure. **a** Port placement. The 12-mm port highlighted in red is an access port for the AirSeal System. **b** Surgical findings. The hepatoduodenal ligament was shortened and the stomach deformed into a horseshoe shape. **c** Scheme of bypass surgery

The repeat sputum PCR test was positive for Mycobacterium tuberculosis. The patient was diagnosed with duodenal TB with duodenal stenosis, tuberculous abdominal lymphadenitis, and pulmonary TB. Due to the difficulty in eating caused by GOO, surgical intervention was prioritized over ATT. The patient underwent laparoscopic bypass surgery. To prevent the airborne transmission of TB bacteria, we employed a closed-circuit insufflation management system (AirSeal® System; CONMED Corporation, Florida, USA). The laparoscopic observation revealed that the area around the hepatoduodenal ligament, antrum, and duodenum D1/D2 was shrunk with redness and the stomach deformed into a horseshoe shape (Fig. 6b). We performed laparoscopic gastrojejunostomy and Braun anastomosis using an automatic stapler (Fig. 6c). The operation time was 211 min, with minimal bleeding.

**Fig. 7:**
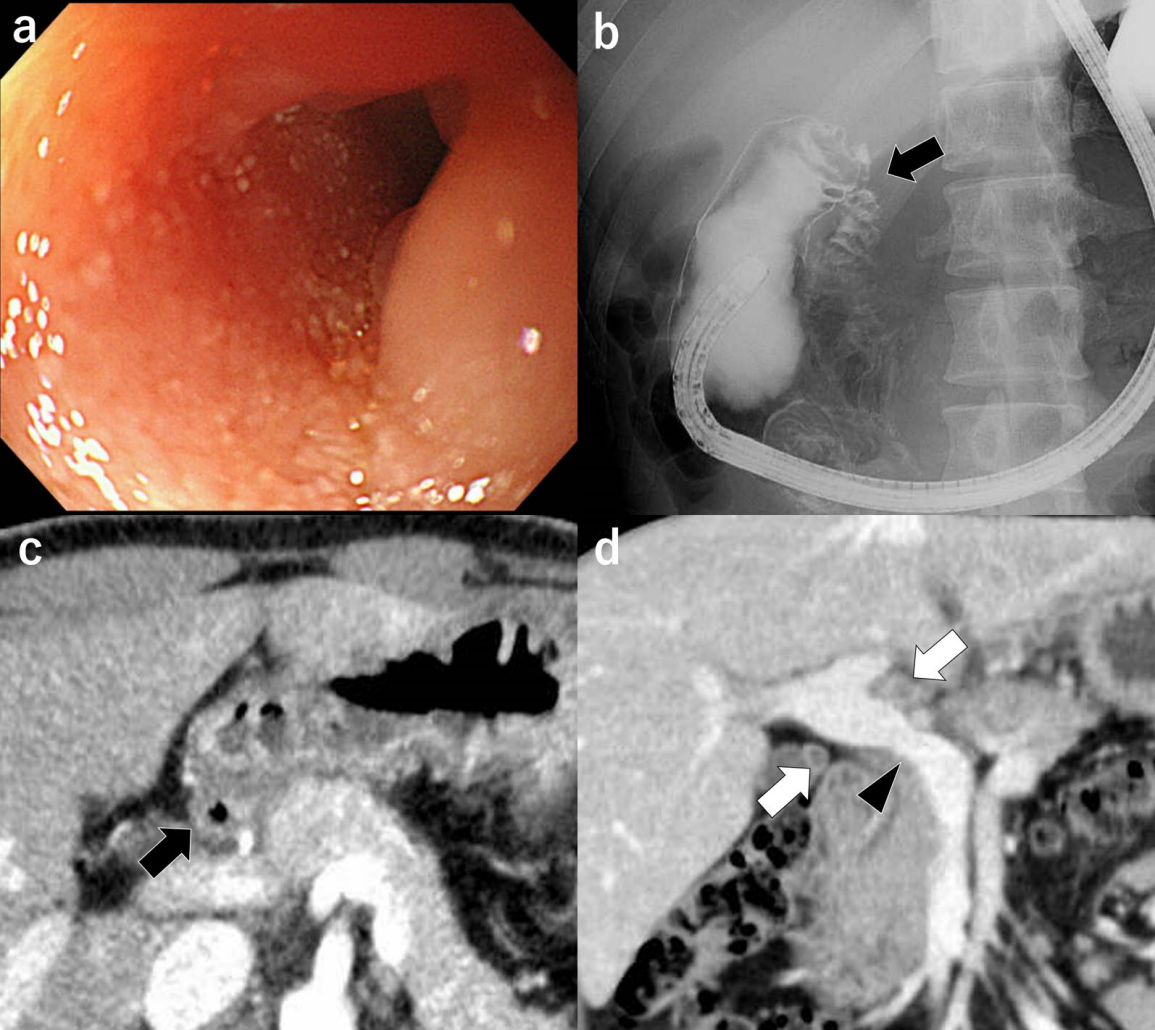
Follow-up images at 6 months after starting antituberculosis treatment. **a** The mucosal edema and stricture of the duodenum have improved slightly. **b** Upper GI also shows residual duodenal stricture (arrow). **c** Edema of the duodenal wall has improved (arrow). **d** There was a slight reduction in lymph nodes (white arrows) and some improvement in portal vein stenosis (head arrow)

The postoperative course progressed smoothly. The patient resumed eating on the 3rd day after surgery and no longer required intravenous fluids on the 4th day. The patient was transferred to a specialized hospital on the 11th postoperative day. Because drug-resistant TB was not detected, ATT was initiated with isoniazid, rifampicin, pyrazinamide, and ethambutol. After receiving ATT for 6 months, follow-up endoscopy and CT were performed. In the follow-up endoscopy, the duodenal stricture showed slight improvement, but the 9.7-mm-diameter scope still could not pass through (Fig. 7a), and upper GI radiography also indicated the presence of residual stenosis (Fig. 7b). CT showed an improvement in the duodenal edema; however, the reduction in the size of the enlarged lymph nodes and improvement in portal vein stenosis were only marginal (Fig. 7c, d). The pulmonary cavitary lesions decreased in size and were mostly scars. To achieve further improvement, isoniazid and rifampicin continued for an additional 3 months. Despite persistent duodenal stricture, the bypass procedure functioned effectively, enabling the patient to eat easily. The patient's body weight showed steady and consistent recovery.

## Discussion

Duodenal TB is a rare form of gastrointestinal tuberculosis (GI TB), accounting for 1–6% of GI TB cases [2]; but it is often reported in the literature. We searched for case reports of duodenal TB with GOO in PubMed using the keywords “duodenal tuberculosis” and “obstruction.” We reviewed 21 cases, including 17 full-text articles [3–19] and our own (Table 1). In this case, it is particularly noteworthy that we report the utility of EUS-FNA in diagnosing duodenal TB. In addition, we demonstrated that laparoscopic bypass surgery is a minimally invasive and safe approach for patients with nutritional deficiencies associated with GOO caused by duodenal TB.

**Table 1:** Details of previous reports and our case

Year, authors	Country of author	Age/sex	Symptoms	Onset time	PTB	Part of lesion	Pre-treatment diagnosis	Treatment (surgical procedure)	Diagnostic method (tool of Bx)	Response to treatment
1985, Ahmed M	Pakistan	22/M	Bowel obstruction, Fever	4 M	(−)	D3	–	Operation → ATT (Duodenojejunostomy)	Pathological (Surgical)	–
		8/M	Vomit, Abdominal pain	Few M	(−)	D3	–	ATT only	Empirical treatment	1 M for weight gain
1986, Kriplani AK	India	45/M	Fullness, Vomit, Weight loss	6 M	(+)	D3	–	Operation → ATT (Gastroenterostomy, Truncal vagotomy)	Pathological (Surgical)	–
1996, Mani S	India	18/M	Epigastric pain, Vomit	6 M	(+)	D2	–	Operation → ATT (Gastroenterostomy, Truncal vagotomy)	Pathological (Surgical)	6 M for symptom free
2005, Rautou PE	French	32/M	Abdominal pain, Esophagitis, Duodenal stenosis	–	(−)	D2	Zollinger–Ellison syndrome	Operation → ATT (Pancreatoduodenectomy)	Pathological (Surgical)	–
2008, Benzekri O	Morocco	60/M	Abdominal pain, Vomit, Weight loss	1 M	(−)	D1	–	Operation → ATT (Gastroenterostomy)	Pathological (Surgical)	11 M for symptom improvement
		57/M	Epigastric pain, Vomit, Weight loss	6 M	(−)	D3	–	Operation → ATT (Duodenojejunectomy, Duodenojejunostomy)	Pathological (Surgical)	3Y for symptom free
2011, Flores HB	Philippines	31/M	Epigastric pain, Vomit	1Y	(−)	D1	Peptic ulcer	Operation → ATT (Gastrojejunostomy)	Pathological (Surgical)	3 M for symptom free
2011, Al-Hilou H	UK	62/F	Heartburn, Chest discomfort, Malaise, Weight loss	9 M	(−)	D2	Suspicious of TB	ATT only	Pathological, Bacteriological (EUS-FNA, Surgical)	–
2012, Chawla I	India	42/M	Epigastric pain, Fullness, Vomit	25D	(−)	D3	–	Operation → ATT (Gastrojejunostomy, Jejunojejunostomy)	Pathological (Surgical)	–
2013, Padmanabhan H	UK	33/M	Dyspepsia, GOO, Weight loss	3Y	(−)	D1, D2	Crohn’s disease or TB or Peptic ulcer	Endoscopic balloon dilation + ATT	Bacteriological (Endoscopic)	3 M for improvement of endoscopic findings
2013, Sisodiya R	India	35/F	Fullness, Vomit, Weight loss	3Y	(−)	D3	SMA syndrome, Duodenal stricture	Operation → ATT (Jejunum resection, Duodenojejunostomy, Ileocecal resection)	Pathological (Surgical)	–
2014, Fatemi SR	Iran	18/F	Abdominal pain, Nausea, Vomit, Fatigue, Powerless, Weight loss	4 M	(−)	D2, D3	Retroperitoneal lymphoma or GIST or Desmoid tumor	Operation → ATT (Gastrojejunostomy)	Pathological, Bacteriological, PCR (Surgical)	–
2017, Kalpande S	India	13/M	Vomit, Fever, Epigastric pain, Weight loss	14D	(−)	D1, D2	Peptic ulcer	Operation → ATT (Gastrojejunostomy)	Pathological (Surgical)	3 M for partial improvement of endoscopic findings
2017, Lee JM	Korea	47/F	Vomit, Weight loss	1 M	(+)	D3, D4	TB	ATT → Operation (Laparoscopic duodenojejunostomy)	PCR (Endoscopic)	–
2018, Udgirkar S	India	24/F	Vomit, Fever, Weight loss	1 M	(−)	D1, D2	TB	ATT only	Pathological, Bacteriological (Endoscopic)	6 M for improvement of endoscopic findings
		22/F	Upper abdominal pain, Vomit	1 M	(−)	D1	TB	ATT only	Bacteriological (Endoscopic)	5 M for improvement of endoscopic findings
2019, Meregildo-Rodríguez E	Peru	31/M	Fullness, Epigastric pain, Nausea, Vomit, Weight loss	4 M	(−)	D2, D3	Duodenal stricture	ATT only	Pathological (Endoscopic)	2 M for symptom improvement
2020, Chang A	Thailand	52/M	Abdominal pain, Early satiety, Weight loss	3 M	(−)	D2	Peptic stricture	Endoscopic balloon dilation → ATT	Bacteriological (Endoscopic)	6 M for some improvement and 12 M for complete resolution of endoscopic findings
2023, Molla YD	Ethiopia	48/M	Vomit, Epigastric pain, Weight loss	1Y	(−)	D2, D3	TB	ATT only	Pathological (Endoscopic)	6 M for symptom improvement
Our case	Japan	35/M	Epigastric pain, Fullness, Vomit, Weight loss	1 M	(+)	D1, D2	TB	Operation → ATT (Laparoscopic gastrojejunostomy)	Pathological (EUS-FNA), Bacteriological (Septum PCR)	Persistent stenosis at 6 M endoscopy

*ATT* Antituberculosis treatment, *Bx* biopsy, *D* Days, *EUS-FNA* Endoscopic ultrasound-guided fine-needle aspiration, *GIST* Gastrointestinal stromal tumor, *GOO* Gastric outlet obstruction, *M* Months, *PCR* Polymerase chain reaction, *PTB* Pulmonary tuberculosis, *SMA* Superior mesenteric artery, *TB* Tuberculosis, *UK* United kingdom, *Y* Years, – Not mentioned

Diagnosing duodenal TB is difficult due to the lack of specific clinical, endoscopic, and radiological features [20]. As shown in Table 1, among the 13 patients who underwent surgery, only one [15] had a confirmed diagnosis of TB before surgery in addition to ours. Therefore, most patients lack an accurate preoperative clinical diagnosis. Several case series from India, a country with a high TB burden, have shown similar patterns. In a case series of 23 patients, all underwent multiple biopsies during endoscopy but only two were diagnosed preoperatively [21]. In other reports of 30 cases of duodenal TB [22] and in five pediatric cases [23], the diagnosis of TB was made only after surgery in all patients. These facts indicate that despite the importance of making an accurate diagnosis for early and appropriate treatment, the diagnosis of duodenal TB is often delayed.

In addition, diagnostic delay may be due to the low accuracy in obtaining adequate tissue samples from the lesion. According to the guidelines [20], a definitive diagnosis of GI TB can be established if any of the following four criteria are present: demonstrating acid-fast bacilli, a positive TB PCR, caseating granulomas, or a positive TB culture on a biopsy specimen. However, diagnosis of duodenal TB from routine biopsy material is rare because tuberculous granulomas are mainly located in the submucosa [2, 20]. Routine biopsies may not collect sufficient tissue from the deeper layers, including the submucosal region. Therefore, EUS-FNA may provide a higher diagnostic sample yield. In our case, the initial endoscopic biopsy only revealed nonspecific inflammation, and the second biopsy showed granulomas with epithelioid cells but was inconclusive. Characteristic necrotic granulomas and acid-fast bacilli were successfully observed in the lymphoid tissue obtained using EUS-FNA, confirming the diagnosis of tuberculosis. Al-Hilou et al. [3] have also demonstrated the advantages of obtaining tissue microbiopsies using EUS-FNA. Additionally, Puri et al. [24] reported a histological diagnostic rate of 92% in patients with gastroduodenal TB by combining endoscopic biopsies with endoscopic mucosal resection. Both reports emphasize the importance of proactive biopsies when TB is highly suspected. In cases with a high index of clinical suspicion and where there is no target for EUS-FNA, a combination of multiple diagnostic methods following the guidelines [20] is necessary. There is no established gold standard for early detection of abdominal TB and no single test is deemed appropriate. Most importantly, to avoid delays in diagnosis, TB should always be considered in the differential diagnosis of unusual gastrointestinal presentations.

GOO is the most common symptom and clinically significant issue in the management of duodenal TB, affecting 60.8% to 84.3% of patients [21, 22, 25]. The mainstay of TB treatment is long-term combination therapy with multiple anti-TB drugs. However, for duodenal TB with GOO, rapid intervention is necessary, considering aspects such as early improvement in the patient’s nutritional status, the route of anti-TB drugs, and the potential for delayed improvement of stenosis due to drug-resistant TB.

GOO treatment includes two main approaches: endoscopic balloon dilation and surgical intervention. Puri et al. [24] reported a high success rate of 92% with endoscopic balloon dilatation of stenotic segments in 13 patients with gastroduodenal TB. However, endoscopic treatment for GOO requires repetitive procedures [26, 27]. Laparoscopic gastrojejunostomy is considered a better approach due to its shorter treatment period and earlier initiation of a regular diet compared to endoscopic therapy.

Relieving GOO before initiating ATT may be appropriate as a treatment strategy for duodenal TB with GOO. Endoscopic mucosal healing has been reported to occur in 81% of cases as early as 2 months after starting ATT and in 100% of cases by 6 months [28, 29]. However, treatment response to ATT varies among patients and is unpredictable. In our case, despite the absence of drug-resistant TB, endoscopy performed in the sixth month after starting ATT revealed a residual duodenal stricture. Lee et al. [15] reported cases where ATT alone was initiated but showed no improvement, necessitating bypass surgery. As demonstrated in these cases, a bypass route allows for unimpeded food intake, even in patients with a poor response to ATT.

## Conclusions

Although duodenal TB is rare, it is important to accurately diagnose it and provide appropriate treatment, especially in patients presenting with GOO. Aggressive biopsies with high suspicion are necessary for diagnosis, and EUS-FNA has proven to be a useful tool in this regard. In patients with strictures, where estimating the therapeutic response to ATT is challenging, and early improvement in nutritional status is desired, laparoscopic bypass surgery is an appropriate choice because of its minimally invasive nature and rapid recovery.

## Data Availability

The datasets used and/or analyzed in the current study are available from the corresponding author upon reasonable request.

## Abbreviations

ATT: Antitubercular therapy
CT: Computed tomography
EUS-FNA: Endoscopic ultrasound-guided fine-needle aspiration
GI TB: Gastrointestinal tuberculosis
GOO: Gastric outlet obstruction
PCR: Polymerase chain reaction
TB: Tuberculosis
